# Effects of Periodic Religious Fasting for Decades on Nutrient Intakes and the Blood Biochemical Profile

**DOI:** 10.3390/nu13113963

**Published:** 2021-11-06

**Authors:** Anatoli Petridou, Nikolaos E. Rodopaios, Vassilis Mougios, Alexandra-Aikaterini Koulouri, Eleni Vasara, Sousana K. Papadopoulou, Petros Skepastianos, Maria Hassapidou, Anthony Kafatos

**Affiliations:** 1 Laboratory of Evaluation of Human Biological Performance, School of Physical Education and Sport Science at Thessaloniki, Aristotle University of Thessaloniki, Thermi, 57000 Thessaloniki, Greece; apet@phed.auth.gr (A.P.); mougios@phed.auth.gr (V.M.); 2 Department of Social Medicine, Preventive Medicine and Nutrition Clinic, School of Medicine, University of Crete, Voutes, 71003 Iraklion, Greece; alexkoulou@yahoo.com (A.-A.K.); kafatos@med.uoc.gr (A.K.); 3 Laboratory of Animal Physiology, Department of Zoology, School of Biology, Aristotle University of Thessaloniki, 54124 Thessaloniki, Greece; evasara@bio.auth.gr; 4 Department of Nutritional Sciences and Dietetics, International Hellenic University, Thermi, 57400 Thessaloniki, Greece; souzpapa@gmail.com (S.K.P.); mnhass@gmail.com (M.H.); 5 Department of Medical Laboratory Studies, International Hellenic University, Thermi, 57400 Thessaloniki, Greece; pskep@otenet.gr

**Keywords:** biochemical profile, macronutrients, elements, nutrient intake, periodic fasting, vitamins

## Abstract

The aim of the present study was to examine differences and correlations in nutrient intakes and serum parameters related to nutrient intake (lipid profile, vitamins, and trace elements) in 200 lifelong Christian Orthodox Church (COC) fasters with periodic abstinence from certain foods (predominantly of animal origin) for approximately half of the year and 200 non-fasting controls, all of whom did not take dietary supplements. Nutrient intakes were assessed through three-day dietary recalls. Blood samples were drawn for the analysis of potential biomarkers of nutrient intake. Fasters had lower energy intake, due to lower fat and protein intake, compared to non-fasters (*p* < 0.05). Fasters also had lower intakes of vitamins A, B_1_, B_2_, B_6_, B_12_, D, folate, pantothenate, sodium, calcium, zinc, and phosphorus. Most participants (in both groups) did not meet the recommended dietary allowances of most vitamins and elements. Most serum biochemical parameters did not reflect the differences in nutrient intakes between groups, and none exhibited a correlation coefficient above 0.5 with nutrient intakes. Our findings suggest that COC fasting is associated with reduced intake of many nutrients, although this does not seem to have an impact on the blood biochemical profile.

## 1. Introduction

Abstinence from certain foods, permanently or periodically, has been practiced by humans for millennia. Examples of permanent abstinence include vegetarianism (in its various nuances), whereas examples of periodic abstinence include religious fasting, as that practiced by faithful followers of the Christian Orthodox Church (COC). These abstain from certain foods, predominantly of animal origin, for periods totaling approximately half of the year [1]. Fasting periods are characterized by high intakes of legumes, cereals, vegetables, and fruits. As such, COC fasting constitutes a distinct dietary pattern, the study of which may offer insight into the relationship between diet and metabolic health.

We recently completed a large project examining the impact of lifelong COC fasting on nutrient intake and various health indicators. We showed that (a) men and women fully adhering to COC fasting for decades, whether being older [1] or younger [2], did not differ in bone mineral density, bone mineral content, or prevalence of osteopenia, osteoporosis, and bone fracture from non-fasting counterparts; (b) abstinence from dairy products and meat does not adversely affect musculoskeletal metabolism or bone density [3]; (c) COC fasters had lower vitamin D status than non-fasting controls, although without impact on bone health [4]; and (d) differences in protein intake from diverse animal and plant sources, as well as in total, had a minor (if any) impact on bone health [5]. 

The aim of the present report is to (a) compare nutrient intakes and serum biochemical parameters between the lifelong COC fasters and non-fasters and (b) examine possible associations between these variables that might provide new information on how diet affects metabolic health.

## 2. Materials and Methods

### 2.1. Study Design and Participants 

This is a cross-sectional study that included 200 fasters (131 women and 69 men) and 200 non-fasters (126 women and 74 men). The fasters had been adhering to religious fasting for a median of 15 years, ranging from 10 to 32 years and with 15 (10–26) years as the starting age of fasting. Details of the study design and the participants have been presented previously [1,2].

### 2.2. Description of COC Fasting 

COC fasting involves abstaining from certain foods—predominantly of animal origin (meat, poultry, eggs, and dairy products), except seafood and snails—during five main periods, three important religious days, Wednesdays, and Fridays, totaling 159 to 197 (average, 178) days per year depending on when Easter falls and does not involve abstaining from food consumption during certain hours of the day, which characterizes intermittent fasting. The COC has set two periods, comprising a total of 47 days, of no food restriction (not even on Wednesdays or Fridays) to avoid nutrients deficiency in the body due to prolonged fasting. Between these extremes (fasting periods and periods of no food restriction) lie 17 to 23 weeks of moderate fasting, that is, only on Wednesdays and Fridays. Therefore, to make the data regarding fasters as representative of the entire year as possible, all measurements, interviews, and blood sampling were performed during these weeks.

### 2.3. Anthropometric Characteristics 

Body weight was measured to the nearest 0.1 kg and height to the nearest 0.01 m on a digital scale with a built-in stadiometer, and body mass index (BMI) was calculated from the measurements. Waist-to-hip ratio was measured using a stretch-resistant tape.

### 2.4. Nutrient Intakes 

Nutrient intakes were assessed through interviewer-based recalls of food consumed over three days, which included a Wednesday or Friday (during which the fasters obeyed fasting), another weekday, and a weekend day. Participants were interviewed about all foods and liquids consumed during those days and the means of the three days are presented. None of the participants in the study received any dietary supplements. Food intake records were analyzed using the Food Processor Nutrition Analysis software (ESHA, Salem, OR, USA). Vitamin and element intakes were compared to their recommended dietary allowances (RDAs) or adequate intakes (AIs), as reported by the Food and Nutrition Board of the Institute of Medicine, National Academy of Sciences [6].

### 2.5. Biochemical Parameters 

Fasting venous blood samples were drawn and treated as described [1]. Serum glucose, triglycerides, total cholesterol, HDL-cholesterol, LDL-cholesterol, urea, creatinine, uric acid, γ-glutamyltransferase, insulin, folic acid, vitamin B_12_, calcium, magnesium, iron, and phosphate were measured in two automatic analyzers, an Abbott Architect i2000SR and a Mindray BS-300, with manufacturers’ kits. The coefficients of variation for all parameters ranged from 1 to 5%, and the laboratory carrying out the analyses participated in a nationwide external quality control program.

### 2.6. Ethical Approval

The study was approved by the Bioethics Committee of the then Alexander Technological Educational Institute of Thessaloniki, presently International Hellenic University (31.5/5679/17-12-2013), and all procedures were in accordance with the Declaration of Helsinki. Each participant was informed about the aims, benefits, and potential risks of the study and provided written informed consent before data collection and blood sampling.

### 2.7. Statistical Analysis

The Kolmogorov–Smirnov test and histogram charts were used to assess normality of distribution. The distribution of almost all variables differed significantly from the normal. Thus, we report all variables as median (interquartile range) and compared groups (fasters vs. non-fasters) by using the non-parametric Mann–Whitney *U* test. Correlation analysis between all variables in the entire sample was performed by determining Spearman’s ρ correlation coefficient. Statistical analysis was performed using the SPSS, version 27 (SPSS, Chicago, IL, USA). All tests and corresponding *p* values were two-sided, and the level of statistical significance was set at α = 0.05.

## 3. Results

### 3.1. Characteristics of Participants

Fasters and non-fasters did not differ in age, body weight, height, BMI, and waist-to-hip ratio (*p* > 0.05). The respective values were 45 (27–58) years vs. 46 (24–57) years, 72.9 (62.9–81.4) kg vs. 71.3 (60.5–84.0) kg, 1.66 (1.60–1.72) m vs. 1.66 (1.61–1.74) m, 26.6 (23.0–29.5) kg/m^2^ vs. 25.6 (22.7–29.1) kg/m^2^, and 0.89 (0.82–0.97) vs. 0.89 (0.81–0.98).

### 3.2. Dietary Intakes

Total daily energy intake was lower in fasters than in non-fasters, that is, 1496 (1256–1825) kcal vs. 1611 (1303–1903) kcal in non-fasters (*p* = 0.027). Macronutrient intakes by the participants are presented in Table 1. The significant differences identified were the lower consumption of fat, saturated and polyunsaturated fatty acids, cholesterol, and protein by fasters compared to non-fasters.

**Figure 1:**
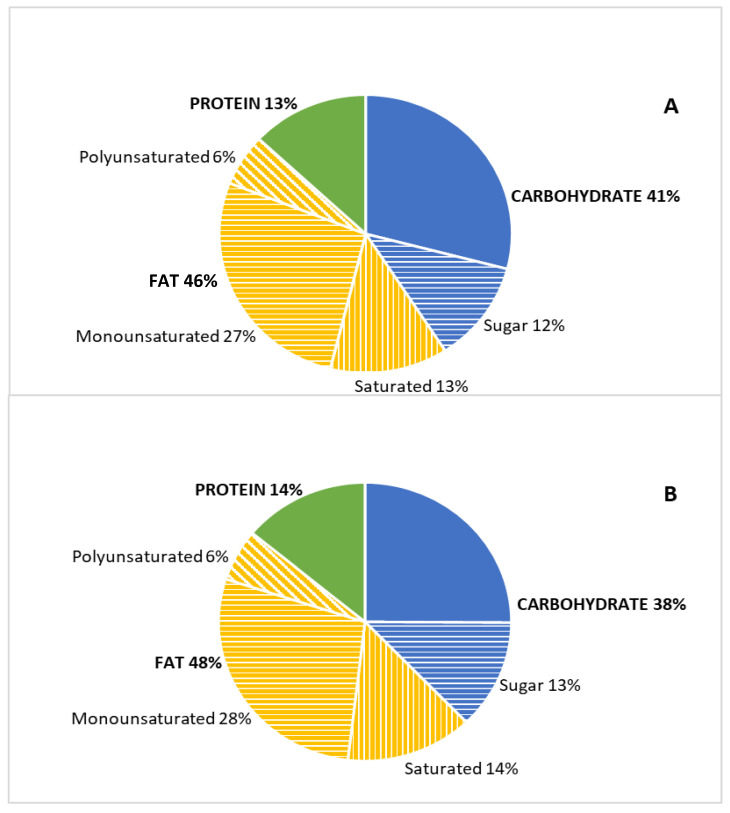
Percentage energy distribution of macronutrients in fasters (**A**) and non-fasters (**B**).

Energy distribution among macronutrients was similar between groups (Figure 1), with fat being the major contributor.

Vitamin and element intakes are shown in Table 2 and Table 3, respectively. The intakes of most vitamins (A, B_1_, B_2_, B_6_, B_12_, D, folate, and pantothenate) and percentage coverage of the corresponding RDAs were significantly lower in fasters compared to non-fasters (Table 2). Most participants (in both groups) did not meet the vitamin RDAs (as can be seen by the fact that the median was below 100%), except for vitamins B_1_, B_2_, and B_12_ in non-fasters, as well as vitamin C in both groups.

Concerning elements, fasters had significantly lower intakes and % RDAs of sodium, calcium, zinc, and phosphorus compared to non-fasters (Table 3). Only phosphorus intake by most participants in both groups was above the RDA, whereas sodium intake was above the RDA by most non-fasters.

### 3.3. Biochemical Profile

The values of the serum biochemical parameters measured are presented in Table 4. Fasters had significantly lower glucose, urea, vitamin B_12_, and phosphate, as opposed to significantly higher insulin, folate, and magnesium compared to non-fasters.

### 3.4. Correlations

A plethora of significant correlations (*p* < 0.05) were detected between the study parameters. To focus on the meaningful ones, we only considered correlations that were moderate or high (ρ > 0.5). Regarding correlations between the serum parameters, phosphate correlated negatively with insulin and folate (ρ = –0.68, *p* < 0.001 for both), whereas no correlation between serum parameters and dietary intakes had a ρ above 0.5. Additionally, there were many moderate or high correlations between dietary intakes, most of which could be explained by the concomitant abundance of certain nutrients in certain foods. For example, protein intake correlated with cholesterol intake (ρ = 0.77, *p* < 0.001), apparently as a result of the abundance of both in meat products, and monounsaturated fatty acids correlated with vitamin E (ρ = 0.61, *p* < 0.001), apparently due to the abundance of both in olive oil. Finally, all macronutrient intakes, some vitamins (vitamin B_1_, vitamin B_2_, vitamin B_6_), and most of the elements (Ca, Fe, Mg, P, Na, K, Zn) were strongly (ρ between 0.52 and 0.84) correlated with total energy intake.

## 4. Discussion

The aim of the present study was to examine whether periodic abstinence from certain foods (mainly of animal origin) for decades, according to the dictates of COC fasting, impacts nutrient intakes and the biochemical profile in relation to metabolic health. When comparing the group of fasters to that of non-fasters, a first observation was that, although the former had lower daily energy intake, they did not differ in indices of fatness (such as BMI and waist-to-hip ratio) from the latter. This could be explained by a difference in energy expenditure, although we have shown that the two groups did not differ in exercise patterns [1,2]. However, the possibility remains that there was a difference in daily activities that they did not perceive as exercise and, hence, did not report as such. Unfortunately, it was not possible to employ more objective means, such as activity trackers, to assess energy expenditure.

It is interesting to note that the lower energy intake by fasters was not due to lower carbohydrate intake but, rather, to lower fat and protein intakes, compared to non-fasters (Table 1). This can be explained by the fasters’ lower consumption of red meat, poultry, eggs, and dairy products, as we have shown in our previous report [5]. The same differences in food group consumption can explain the lower cholesterol intake by the fasters (Table 1). Nevertheless, none of the parameters of the lipidemic profile differed between groups (Table 4) or correlated with any fat intake parameter, in agreement with the tenet that dietary fat intake has little, if any, influence on the lipidemic profile [7,8].

A striking preponderance of fat as an energy source (nearing 50% in both groups, Figure 1) was in stark contrast to the recommendation for 20–35% of total energy [8]. We and others have repeatedly shown increased fat consumption by Greeks (e.g., references [9,10,11]). Luckily, this is due in large part to high olive oil consumption, as evidenced by the dominance of monounsaturated fatty acids (27 to 28% of total energy intake). Nevertheless, energy intake from saturated fatty acids exceeded the recommended 10% [8] in both groups.

Contrary to the lack of any association of the lipidemic profile with the fat intake parameters, the serum urea concentration (Table 4) did reflect the lower protein intake by the non-fasters (Table 1). This is in agreement with the acceptance of serum urea as an index of dietary protein intake [12] and suggests that the former can be used as a surrogate for the latter.

Two indices of glucose homeostasis, that is, the serum glucose and insulin concentrations, exhibited opposite differences between groups. In particular, glucose was higher in non-fasters, whereas insulin was higher in fasters (Table 4). However, the values of both parameters were relatively low, and we do not see any clinical significance in their differences.

The lower energy intake by the fasters was accompanied by a lower intake of most of the vitamins and some elements, compared to non-fasters (Table 2 and Table 3). This included folate, which may seem counter-intuitive, since fasters are expected to consume more fruits and vegetables than non-fasters. However, the lower levels of folate in fasters could be explained by their lower consumption of meats (as we have shown in ref. [5]) since, contrary to common perception, meats are a better source of folate than fruits and vegetables [13].

It is noteworthy that vitamin and element intakes were generally low, a problem that other studies have also highlighted [9,14,15,16,17], although, contrary to ours, those studies did not exclude persons who consumed dietary supplements. It appears that food quality was rather poor, resulting in low nutrient density. This is in agreement with our previous finding of moderate adherence to the Mediterranean diet by both groups [1,2]. However, this plethora of shortfall nutrients did not seem to have an impact on most of the related biochemical parameters that we had the financial resources to measure (that is, vitamin B_12_, calcium, magnesium, iron, and phosphate), since most participants had values within the corresponding reference intervals. By contrast, most participants had a serum concentration of folate below its lower reference limit (3.1 ng/mL), apparently as a consequence of its low intake by both groups.

The absence of an effect of nutritional inadequacies on most of the biochemical parameters tested is in accordance with our previous findings of no effect of inadequate calcium intake on bone health in the same sample [1,2]. Possible reasons for the fact that some biochemical parameters do not reflect nutritional inadequacies are metabolic homeostatic mechanisms that compensate for reduced nutrient intake by increasing intestinal absorption, increasing efflux from tissues into the blood, and/or decreasing excretion.

A problem frequently encountered in nutritional studies that are based on self-reported food intake is misreporting (and usually underreporting), which is considered unavoidable [18]. To assess misreporting in the present study, we compared the total daily energy intake by the participants, as assessed through their dietary records, to the dietary reference values for energy of the European Food Safety Authority [19]. Our calculations showed a moderate underreporting of 18%. By correcting the micronutrient intakes for this underreporting, we found that the percentage coverage of the RDAs would exceed 100% in seven cases (vitamin B_1_, vitamin B_2_, vitamin B_12_, Na, and Fe in fasters; and vitamin B_6_ and Fe in non-fasters) in which it was previously below 100%. This, however, does not change the conclusion that the intakes of most micronutrients were below their RDAs. 

Of interest is the negative correlation of serum phosphate with insulin found in the present study, since it agrees with the finding that, in healthy subjects, low serum phosphate was associated with reduced insulin sensitivity [20]. As is the case with association studies, the authors were unable to establish a cause and effect, that is, whether low phosphate is a cause or a consequence of low insulin sensitivity.

The dearth of correlations between serum biochemical parameters and nutrient intakes in our work is in accord with the few studies that have addressed the same issue or the correlation of biochemical parameters with dietary habits [21,22,23]. Indeed, these authors have found a limited number of correlation coefficients above 0.5. It seems that nutrient metabolism is so complex and versatile that more intense research efforts are needed in the direction of discovering valid and reliable biomarkers of the composition of our diet.

## 5. Conclusions

Faithful followers of COC fasting had lower intakes of total daily energy, fat, and protein during periods of moderate fasting (that is, between periods of strict fasting and periods of no food restriction), periods that we consider representative of the whole year, compared to non-fasting controls. Fasters had also lower intakes of most vitamins and some elements. Most participants (in both groups) did not meet most of the vitamin and element RDAs. Most serum biochemical parameters did not reflect the differences in nutrient intakes between groups, and none exhibited a meaningful correlation with nutrient intakes.

## Figures and Tables

**Table 1:** Daily macronutrient intakes by fasters and non-fasters, based on three-day dietary records (median and interquartile range).

Macronutrient	Fasters (*n* = 200)	Non-Fasters (*n* = 200)	*p*
Carbohydrate (g)	156.8 (124.6–180.7)	155.2 (119.9–194.3)	0.994
Sugar (g)	44.4 (29.2–61.6)	51.4 (29.3–65.5)	0.217
Dietary fiber (g)	19.0 (15.6–25.4)	19.5 (14.0–25.3)	0.949
Fat (g)	76.8 (62.0–98.6)	85.9 (68.3–103.5)	0.009
Saturated fatty acids (g)	20.0 (13.7–26.6)	22.4 (18.3–31.0)	<0.001
Monounsaturated fatty acids (g)	40.8 (30.7–51.3)	43.8 (33.1–54.4)	0.159
Polyunsaturated fatty acids (g)	8.2 (6.8–10.8)	9.3 (7.0–12.2)	0.019
ω3 Fatty acids (g)	0.5 (0.4–0.8)	0.6 (0.4–0.8)	0.456
ω6 Fatty acids (g)	4.8 (3.0–6.5)	4.5 (2.9–7.2)	0.888
*trans*-Fatty acids (g)	0.4 (0.3–0.9)	0.5 (0.3–0.9)	0.545
Cholesterol (mg)	129.3 (83.1–193.1)	158.8 (115.2–219.5)	<0.001
Protein (g)	47.5 (40.9–62.5)	56.5 (44.7–69.1)	<0.001
Protein (g/kg body mass)	0.70 (0.51–0.87)	0.76 (0.63–0.96)	0.002

**Table 2:** Daily vitamin intakes by the two groups, based on three-day dietary records (median and interquartile range).

Vitamin	Fasters (*n* = 200)	Non-Fasters (*n* = 200)	*p*
Vitamin A (RE)	286 (184–491)	344 (211–623)	0.008
Vitamin A (% RDA)	38.0 (24.0–62.5)	46.0 (27.3–82.8)	0.007
Vitamin B_1_ (mg)	1.02 (0.78–1.37)	1.14 (0.84–1.55)	0.048
Vitamin B_1_ (% RDA)	91.0 (71.0–121.0)	101.5 (76.0–133.0)	0.040
Vitamin B_2_ (mg)	1.01 (0.75–1.38)	1.23 (0.90–1.72)	<0.001
Vitamin B_2_ (% RDA)	87.0 (62.3–118.8)	107.0 (78.0–148.5)	<0.001
Niacin (mg)	8.36 (5.18–12.04)	8.98 (6.18–12.94)	0.093
Niacin (% RDA)	56.0 (36.0–83.0)	61.5 (43.3–86.0)	0.081
Vitamin B_6_ (mg)	1.10 (0.88–1.46)	1.35 (0.96–1.76)	<0.001
Vitamin B_6_ (% RDA)	78.0 (63.0–103.0)	94.0 (66.0–120.8)	<0.001
Vitamin B_12_ (µg)	2.01 (0.87–2.97)	2.45 (1.31–4.04)	<0.001
Vitamin B_12_ (% RDA)	83.5 (36.0–124.0)	103.0 (55.0–168.0)	<0.001
Vitamin C (mg)	109.5 (58.3–178.3)	99.5 (56.1–146.5)	0.098
Vitamin C (% RDA)	138.0 (77.0–224.5)	117.0 (71.3–191.8)	0.074
Vitamin D (µg)	1.25 (0.45–2.73)	1.74 (0.59–3.18)	0.026
Vitamin D (% RDA)	8.0 (3.0–18.0)	12.0 (4.0–21.0)	0.017
Vitamin E (mg)	5.91 (3.88–7.30)	5.71 (3.58–8.68)	0.312
Vitamin E (% RDA)	39.5 (26.0–49.0)	38.0 (24.0–57.8)	0.315
Folate (µg)	169 (117–219)	195 (138–267)	<0.001
Folate (% RDA)	42.0 (29.0–55.0)	48.5 (34.3–66.8)	<0.001
Pantothenate (mg)	1.96 (1.37–2.61)	2.20 (1.62–3.26)	0.003
Pantothenate (% AI)	39.5 (27.3–52.0)	44.0 (32.3–65.0)	0.003

AI, adequate intake; RDA, recommended dietary allowance; RE, retinol equivalent.

**Table 3:** Daily element intakes by the two groups, based on three-day dietary records (median and interquartile range).

Element	Fasters (*n* = 200)	Non-Fasters (*n* = 200)	*p*
Na (mg)	1450 (1051–1948)	1655 (1204–2180)	0.006
Na (% AI)	96.5 (70.0–130.0)	110.5 (80.0–145.8)	0.006
K (mg)	1837 (1524–2217)	1835 (1407–2296)	0.932
K (% AI)	62.0 (52.0-78.0)	63.0 (48.0–81.8)	0.781
Ca (mg)	531.7 (349.9–761.3)	658.5 (455.0–884.0)	<0.001
Ca (% RDA)	51.0 (33.0–72.0)	61.5 (43.0–82.8)	<0.001
Mg (mg)	165.5 (127.7–205.8)	168.4 (126.8–220.6)	0.395
Mg (% RDA)	47.0 (37.0–60.0)	50.5 (37.0–61.8)	0.519
Fe (mg)	9.12 (7.47–11.19)	9.27 (7.31–12.64)	0.354
Fe (% RDA)	96.0 (63.0–125.5)	96.5 (60.0–142.5)	0.461
Cu (mg)	0.61 (0.44–0.79)	0.65 (0.43–0.86)	0.298
Cu (% RDA)	67.5 (49.0–88.0)	72.0 (48.3–95.5)	0.298
Zn (mg)	5.77 (4.00–7.95)	7.32 (5.29–9.44)	<0.001
Zn (% RDA)	64.0 (45.3–85.0)	80.0 (60.0–111.0)	<0.001
Mn (mg)	1.04 (0.69–1.68)	1.07 (0.73–1.64)	0.610
Mn (% AI)	52.0 (35.3–86.8)	56.5 (36.3–83.5)	0.688
P (mg)	727.3 (586.9–920.0)	840.1 (651.3–1097.3)	<0.001
P (% RDA)	104.0 (84.0–131.5)	120.0 (93.0–156.5)	<0.001
Se (µg)	41.02 (26.68–63.42)	43.44 (29.72–64.45)	0.354
Se (% RDA)	74.5 (48.3–115.0)	79.0 (54.0–117.5)	0.361

AI, adequate intake; RDA, recommended dietary allowance.

**Table 4:** Serum biochemical parameters of participants in the two groups (median and interquartile range).

Parameter	Fasters (*n* = 200)	Non-Fasters (*n* = 200)	*p*
Glucose (mg/dL)	81 (73–89)	85 (77–93)	0.004
Triglycerides (mg/dL)	103 (73–160)	109 (83–191)	0.157
Total cholesterol (mg/dL)	186 (154–215)	188 (160–229)	0.117
HDL-cholesterol (mg/dL)	53 (42–6)	53 (44–66)	0.540
LDL-cholesterol (mg/dL)	101 (73–142)	96 (77–137)	0.941
Urea (mg/dL)	277 (217–337)	307 (247–37)	0.001
Creatinine (mg/dL)	0.91 (0.83–1.03)	0.94 (0.83–1.06)	0.102
Uric acid (mg/dL)	4.3 (3.5–5.3)	4.5 (3.7–5.5)	0.343
γ-Glutamyltransferase (U/L)	16 (12–23)	15 (12–22)	0.759
Insulin (µIU/m)	3.1 (1.3–5.6)	2.1 (1.2–4.2)	0.007
Folate (ng/mL)	2.3 (0.9–4.5)	1.4 (0.9–3.1)	0.004
Vitamin B_12_ (pg/mL)	287 (221–373)	315 (249–3979)	0.022
Calcium (mg/dL)	9.7 (9.4–10.1)	9.7 (9.5–10.0)	0.824
Magnesium (mg/dL)	1.88 (1.76–2.01)	1.80 (1.63–1.94)	<0.001
Iron (µg/dL)	98 (71–121)	91 (67–120)	0.251
Phosphate (mg/dL)	4.6 (3.6–6.4)	5.7 (4.5–6.7)	<0.001

## Data Availability

Informed consent was obtained from all subjects in the study.
